# Midterm prognosis and surgical implication for clival chordomas after extended transsphenoidal tumor removal and gamma knife radiosurgery

**DOI:** 10.1186/s12883-021-02234-4

**Published:** 2021-05-22

**Authors:** Yoshikazu Ogawa, Hidefumi Jokura, Teiji Tominaga

**Affiliations:** 1 Department of Neurosurgery, Kohnan Hospital, 4-20-1 Nagamachi Minami, Taihaku-ku, Sendai, Miyagi 982-8523 Japan; 2 Jiro Suzuki Memorial Gamma House, Furukawa Seiryo Hospital, Osaki, Miyagi Japan; 3 Department of Neurosurgery, Tohoku University Graduate School of Medicine, Sendai, Miyagi Japan

**Keywords:** Chordoma, Gamma knife radiosurgery, Maximal removal, Prognosis, Transsphenoidal approach

## Abstract

**Background:**

Treating chordoma through surgery alone is often ineffective. Thus, surgery often performed with irradiation, with a reported 5-year survival rate of 60–75%. The clinical course varies, and disease rarity prevents larger number of clinical investigations.

**Methods:**

In total, 19 patients with clival chordomas were retrospectively extracted from our institutional database. They were initially treated with maximal tumor removal using the extended transsphenoidal approach between March 2006 and January 2021. When total tumor removal was achieved, prophylactic irradiation was not performed. If tumor remnants or recurrence were confirmed, Gamma Knife (GK) radiosurgery was performed. The mean follow-up period was 106.7 months (ranged 27–224 months). The clinical course and prognostic factors were investigated.

**Results:**

Total removal was achieved in 10 patients, whereas 4 patients suffered recurrence and required GK. GK was applied to 11 patients with a 50% isodose of 13–18 Gy (mean: 15.4 Gy), and eight patients remained progression free, whereas three patients suffered repeated local recurrence and died of tumor-related complications. The mean overall progression-free interval was 57.2 months (range: 6–169 months). One male patient died of tumor un-related lung cancer 36 months after the initial treatment, and other patients survived throughout the observational periods. The mean overall survival was 106.7 months (range: 27–224 months). Thus, the 5-year survival rate was 94.7%. Statistical analysis indicated that sex (men), > 15 Gy of 50% isodose by GK, and screening brain examinations as prophylactic medicine were significant favorable prognostic factors.

**Conclusions:**

The favorable outcomes in this investigation suggest the importance of early detection and treatment. Surgery may enable better conditions for sufficient GK doses.

## Background

Chordomas arise from the prenatal remnants of the notochord and are frequently located on the midline of the skull base, cervical vertebrae, and sacrum [3]. The incidence rate is only 0.5% among all intracranial tumors and 1–4% among skeletal tumors [1, 16, 19, 22]. This rarity of the disease has prevented extensive clinical investigations, causing a great discrepancy between the indolent pathological features and the malignant clinical course [5, 7–10, 12–15], with a reported 5-year survival rate of 60–75%. Although the tumor doubling time is rather long for chordomas, repeated local recurrences and dissemination are not unusual because of the invasive growth along the bone marrow [2, 5]. Tumor control by surgery alone is considered difficult, and thus, most patients are treated using combined therapy with irradiation [6–9, 14, 17]. Because this tumor usually exhibits resistance to conventional radiation therapy and evidence for effective chemotherapy has not yet been established, ion beam therapy including proton or heavy ion beam therapy is expected to provide good tumor control [6, 8, 17]. However once again, the rarity of this disease hinders the accumulation of clinical experiences, and health insurance barely covers treatments with ion beam therapy.

Here, we present a single-center experience of maximal tumor removal of clival chordomas using the extended transsphenoidal approach following Gamma Knife (GK) radiosurgery for visualized remnants or tumor recurrence. The role and the implications of surgery are also discussed.

## Methods

### Patients and surgical approach

This report included 14 men and 5 women aged 41–85 years (mean: 60.4 years) suffering from clival chordomas were included with histological confirmation at the Department of Neurosurgery, Kohnan Hospital between March 2006 and January 2021. The patients were initially treated with maximal tumor removal using the extended transsphenoidal approach with simultaneous removal of the surrounding bone cortex and bone marrow as far as possible within the technical range of skull base repair. Reconstruction of the skull base was performed with autologous fascia tightly sutured to the dural edge of the skull base, fortified with epidural attachment of muscle pieces, and the entire surface of the dural window was wrapped with mucosal flap of the sphenoidal sinus. No type of marsupialization was performed.

### Assessment

All patients underwent axial, coronal and sagittal T1-and T2-weighted magnetic resonance (MR) imaging with and without contrast medium (Signa Horizon, General Electric, Milwaukee, WI; 3.0 Tesla system) and bone image computed tomography (CT) (Discovery CT 750 HD, General Electric) preoperatively and just after the operation. Follow-up MR imaging was performed postoperatively for all patients at 6-month intervals after the operation (1.5 Tesla system; Magnetom, Siemens AG, Erlangen, Germany). When gross total removal was achieved, prophylactic irradiation was not performed, and patients were simply observed at 6-month intervals. If tumor remnants were visualized, the GK radiosurgery was applied upon skull base regeneration (usually approximately 3 months after the operation). When tumor recurrence was confirmed, GK radiosurgery was subsequently applied to the visualized tumor bulk; this occurred in all patients except for one female patient (case 16), who was treated with postoperative fractionated irradiation because of her older age (85 years). All the patients could be followed up throughout their clinical course with MRI evaluation for 27–224 months (mean: 106.7 months).

Gross total removal was defined as the absence of visible tumor bulk on both during intraoperative findings and postoperative MR imaging.

### Radiosurgery planning

**Fig. 1:**
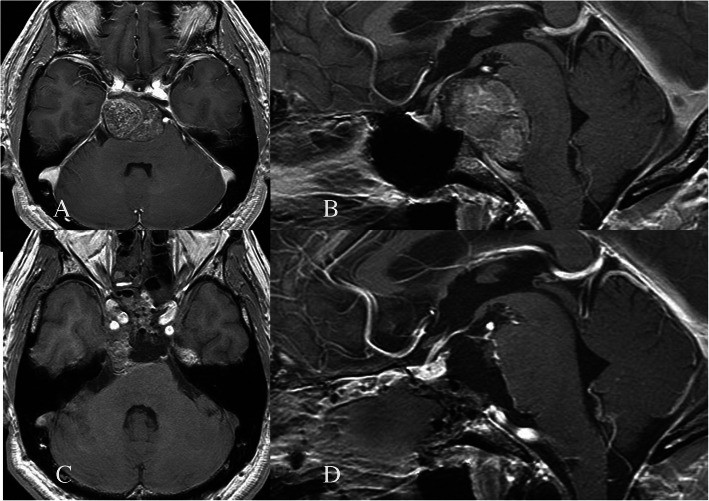
Head MR imaging with contrast medium showing massive clival tumor compressing the entire pons backward (**a**: axial image, **b**: sagittal image); surgery removed 90% of the tumor was removed except for the right retrocavernous sinus portion (**c**: axial image **d**: sagittal image)

In total, 19 GK treatments were performed for residual or recurrent tumors in 11 patients using four generations of GK (Type B (*n* = 1 treatment), Type C with APS (n = 1), Perfexion (*n* = 12) or Icon (*n* = 5). Except for one hypo-fractionated treatment (the sixth treatment of case 1), all other treatments were performed in a single fraction. For dose planning, thin-sliced gadolinium-enhanced T1 and T2 MR images, and heavy T2 images with bone-window CT were used to delineate tumor boarders including the area of bone erosion by the invading tumor (Fig. 1). No margin was added to the tumor margin. The treated tumor volume and prescribed marginal dose were 13–18 Gy using the 50–90% isodose line (Table 2). We attempted to apply at least 16 Gy to the margin of the tumor, however, in some cases this dose had to be decreased because of the proximity of the optic apparatus, prior radiation, or large tumor volume.

### Pathological examination

The surgical specimens were immediately fixed for histology and immunohistochemistry with 10% buffered formalin, then embedded in paraffin, and cut into 3-μm-thick serial sections. Hematoxylin and eosin as well as periodic acid-Schiff staining were performed in all cases. The avidin-biotin-peroxidase complex method was applied for immunohistochemical staining, and cell proliferation was assessed with Ki-67 (MIB-1, Dako, 1:100). Immunohistochemically positive cells were counted within at least 1000 background cells in three high-power visual fields including the hot spot and other fields, and then indicated as a percentage. Normal lymph nodes were used as positive controls for Ki-67.

### Statistical analysis

Statistical comparisons were made using Statmate 5 software (ATMS Co., Ltd., Tokyo, Japan), and *P* values of < 0.05 were considered significant. Univariate analysis between groups was performed. Age, sex, preoperative tumor volume, maximum tumor diameter, total removal (yes or no), Ki-67 labeling index (more or less than 10%), 50% isodose of GK (yes or no) and screening brain examination (yes or no) were compared with log-rank analysis. Any variables with a *P* value of < 0.05 were considered to be potential independent variables and were entered into the multivariate analysis using the Cox proportional hazard model.

### Ethics approval and consent to participate

The surgical policy was explained preoperatively to the patients and written informed consent was obtained, and the study design was approved by the internal ethics committee of Kohnan Hospital 2021.

## Results

Among the 19 patients enrolled in this study, the most common initial symptom was diplopia due to cranial nerve palsy (*n* = 6 patients), optic nerve dysfunction (*n* = 2), and spontaneous cerebrospinal fluid leakage due to clival erosion with no visible tumor (*n* = 1). Overall, 8 patients were asymptomatic, and the tumors were detected using screening brain examinations as prophylactic medicine. Initial treatments were applied in 17 patients; however, the remaining 2 patients had suffered from re-growth after step-wise tumor removal and radiation therapy (Table 1). All of the tumors extended along the clivus, and eight patients experienced extra-arachnoidal localization, whereas the remaining 11 experienced tumor extension into the subarachnoid spaces.

**Table 1:** Preoperative clinical profile

Case	Age Ranges	Sex	Tumor volume (mL)	Maximum diameter (mm)	Initial symptom
1	61–70	F	15.1	39	6 Nerve palsy
2	41–50	M	0.73	12	Brain examination
3	51–60	M	36	50	1 TO, 3GK
4	61–70	M	12.7	40	6 Nerve palsy
5	51–60	M	14.4	32	Incidental
6	41–50	M	9.8	39	1 Removal, 1 FR
7	61–70	M	26.4	50	Nasal congestion
8	41–50	M	0.98	15	Incidental
9	51–60	M	Not visible	Not visible	CSF leakage
10	41–50	M	11.7	30	Brain examination
11	71–80	M	2.52	28	3,4 Nerve palsy
12	71–80	M	5.13	27	6 Nerve palsy
13	61–70	M	3.4	20	Brain examination
14	61–70	F	12.8	41	6 Nerve palsy
15	51–60	F	2.76	23	Brain examination
16	81–90	F	17.9	42	Unilateral blindness
17	51–60	M	68.5	52	Bitemporal hemianopsia
18	51–60	F	66.5	63	3,6 Nerve palsy
19	51–60	M	0.17	8	Brain examination

*F* female, *M* male, *TO* transoral surgery, *GK* gamma knife, *FR* fractionated irradiation

*CSF* cerebrospinal fluid

Gross total removal was achieved in 10 patients, and tumor remnants were observed in nine patients, indicating a removal rate of 73–100% (mean: 92.8%). The tumor recurrence developed in four out of 10 patients with gross total removal, which required GK treatment thereafter. The remaining six patients were observed only, and all of them were asymptomatic preoperatively. Postoperative follow-ups continued for 27–224 months (mean: 106.7 months), the overall progression-free intervals were 6–169 months (mean: 57.2 months), and the overall survival was 27–224 months (mean: 106.7 months), except for one male patient, who died of tumor-unrelated lung cancer 36 months after the initial treatment (case 12). Thus, the 5-year survival rate was 94.7%.

Preoperative tumor exhibited volumes of 0.17–68.4 mL (mean: 16.2 mL) and maximum diameters of 8–63 mm (mean: 32.2 mm). Postoperative residual tumors exhibited volumes of 0–6.17 mL (mean: 1.61 mL). GK radiosurgery was performed 19 times for 11 patients with a 50% isodose of 13–18 Gy (mean: 15.4 Gy). Overall, 8 out of 11 patients remained progression free, whereas 3 patients suffered repeated local recurrence and died of tumor-related complications. The Ki-67 labeling index ranged from less than 1–25.1% (mean: 8.91%). The clinical data are shown in Table 2.

**Table 2:** Postoperative course

Case	Removal rate (%)	Tumor volume at GK (ml)	Marginal dose (Gy)	Isodose (%)	PF period (months)	Overall survival (months)	Treatment and clinical course
1	100	6.6, 3.1,1.0, 1.1, 7.7, 3.2	14, 14, 15, 15, 14, 5 × 5	50, 50, 50, 50, 50, 50	12	186	TS - GK × 6 -
2	100	N/A	N/A	N/A	169	169	TS -
3	75	1.2	20	90	51	224	TS – GK – GK – GK - TS - GK – TC - CK died from disease progression to sarcoma
4	100	7.3, 17.6, 27.8	14, 14, 15	50, 50, 50	48	62	TS - GK - GK - TS - TS - GK died from epistaxis
5	90	2.7	16	50	6	165	TS – TS – GK
6	70	13.0, 3.2	13, 12	50	31	100	TS - FR(50Gy) - TS - GK - TS - GK died from brain stem invasion
7	100	N/A	N/A	N/A	69	150	TS -
8	100	N/A	N/A	N/A	152	152	TS -
9	100	N/A	N/A	N/A	138	138	TS -
10	100	1.0	18	52	95	130	TS – GK
11	100	2.3	16	55	30	127	TS – GK
12	95	4.5	17	50	38	38	TS – GK died from lung cancer
13	100	N/A	N/A	N/A	115	115	TS -
14	80	14.5	16	50	21	62	TS – GK
15	95	1.7	16	50	24	60	TS – GK
16	73	N/A	N/A	N/A	15	51	TS -
17	91	17.6	15	50	19	45	TS – GK
18	95	N/A	N/A	N/A	27	27	TS -
19	100	N/A	N/A	N/A	27	27	TS -

*PF* progression free, *GK* gamma knife, *TC* transcranial surgery, *TS* transsphenoidal surgery, *CK* cyber knife

*N/A* not applicable

The results show that male patients (*p* < 0.01), who received > 15 Gy of 50% isodose at the time of GK radiosurgery (*p* < 0.05), and who received screening brain examination as prophylactic medicine (*p* < 0.05) had a significantly favorable prognosis according to univariate statistical analysis. Gross total removal and a tumor volume of < 1 mL before GK radiosurgery did not appear to have a significant effect on prognosis. An age of > 60 years, a tumor volume of > 10 mL, and a maximum tumor diameter of > 30 mm were not adverse prognostic factors. No statistical significance was observed between patients with Ki-67 labeling indexes of either > 10% or < 10% (Table 3). Subsequent multivariate statistical analysis showed significance in male patients (*p* < 0.02), however, the 95% confidence interval was relatively wide), but not for GK values of > 15 Gy of 50% isodose (*P* = 0.05) or screening brain examination (*P* = 0.08). (Table 4).

**Table 3:** Prognostic factors of progression free (log-rank analysis)

Significant	
Male patient, *p* < 0.01	
50% isodose > 15 G, *p* < 0.05	
Screening brain examination, *p* < 0.05	
Not significant	
Age < 60 years, *p* = 0.72	
Initial tumor volume < 10 ml, *p* = 0.12	
Tumor maximum diameter < 30 mm, *p* = 0.44	
Total removal, *p* = 0.79	
Ki 67 labeling index < 10%, *p* = 0.32

**Table 4:** Multivariate analysis using Cox proportional hazard model

Variables	PE	SE	Wald chi-squared	Precision p value	Risk ratio	95% CI
Sex	2.260	0.925	5.968	0.014	9.591	1.563–58.834
GK > 15 Gy	−0.285	0.150	3.606	0.057	0.751	0.560–1.009
Brain examination	1.212	0.705	2.955	0.085	3.361	0.843–13.394

*GK* gamma knife, *PE* parameter estimates, *SE* standard error, *CI* confidence interval

### Illustrative case (case 5)

**Fig. 2:**
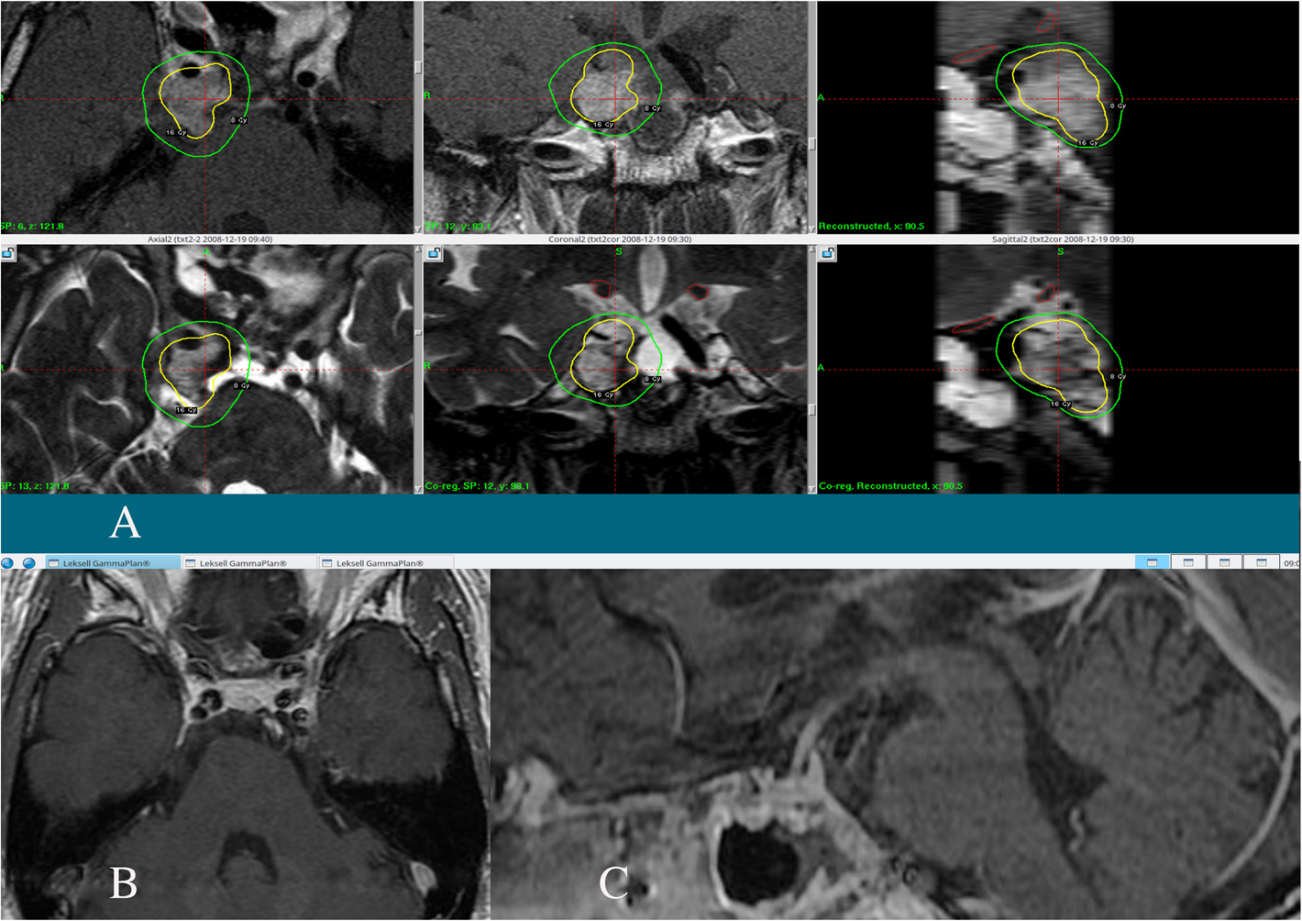
GK radiosurgery was planned with a marginal dose of 16 Gy. The yellow line indicating 16 Gy, and the green as 8 Gy **a**. Follow-up MR imaging revealed complete remission of the tumor (**b**: axial image, **c**: sagittal image)

A 55-year-old man was admitted to the attendant neurosurgical department with continuous nasal obstructive feeling. Head MR imaging showed a massive clival tumor compressing the entire pons backward, and he was introduced to our institute. Extended transsphenoidal surgery was performed in a step-wise manner, resulting in 90% of tumor removal without any neurological deficit (Fig. 1). GK radiosurgery was performed 6 months later to the tumor remnants in the right retrocavernous sinus portion with a marginal dose of 16 Gy (Fig. 2a). He was followed up in 6-months intervals, and complete remission was achieved. Since then, 151 months have passed with no evident tumor recurrence (Fig. 2b, c).

## Discussion

According to one meta-analysis for chordomas, the 5-year survival rate of patients is 60–75%, and treatment results have shown little improvement, even with modern surgical procedures [12]. Although the 5-year survival rate in this investigation was 95% (slightly higher than previously reported) and with no postoperatively acquired neurological deficits, total removal was not a prognostic factor. As such, adjuvant irradiation of this tumor has been confirmed to be essential once again. However, this tumor usually resists conventional radiation therapy with low-dose irradiation to a wide radiation field [6–9, 14, 17]. In contrast, preoperative tumor volume, maximum tumor diameter, however, applying > 15 Gy of a 50% isodose had a significant effect, as a previous report has suggested [11]. Although objective surgical goals vary between reports and the ideal relationship between surgery and postoperative GK radiosurgery is unclear [7, 9, 13, 23], the results in this investigation may suggest the importance of reducing maximal tumor volume. This maximal tumor volume reduction provides an adequate condition for sufficient irradiation, including securing a distance from the optic pathway. Accordingly, we attempted to remove the tumor bulk together with the surrounding bone cortex and bone marrow as far as possible within the technical range of skull base repair. Such expansive removal could contribute to dose escalation of irradiation and/or localization of the radiation field.

Screening brain examination was also identified as another prognostic factor, which was applied as prophylactic medicine mainly targeted to un-ruptured cerebral aneurysms or asymptomatic arterial stenosis. Because the efficacy of this approach across a larger population and the costs vary greatly between nations, this system has not become popular in many countries except for Japan. However, the favorable outcomes in this investigation may indicate the importance of early detection and early treatment of this malignant disease. If a tumor is detected, a no wait-and-see approach should be adopted, rather, early surgery should be implemented even for asymptomatic lesions. The extended transsphenoidal approach is considered less invasive and therefore a better approach for treatment.

Pathophysiological investigations revealed the Ki-67 labeling index, p53 overexpression, and receptor tyrosine kinase expression as possible indicators [4, 20, 21]. Another report presumed a prognostic difference between chondroid chordoma and chordoma [18]. No significant differences were found between these parameters in this investigation (data not shown); however, this may be due to the small sample number or differences of treatment protocols.

The limitation of this investigation was the small patient number. Past reports have compared prognosis by conducting meta-analyses of different disease backgrounds and different treatment protocols, including proton beam irradiation. The reported investigation for chordomas and chondrosarcomas using proton and/or carbon beam irradiation showed 2-year progression free survival of 76.8% and overall survival of 87.2% [6]. Another proton or carbon beam irradiation showed 5-year progression free survival of 81% and overall survival of 86% [17]. To establish standardized treatment guidelines, prospective and larger multi-institutional studies are strongly encouraged.

## Conclusions

The favorable outcomes in this investigation may indicate the importance of early detection and treatment. Surgery may enable favorable conditions for sufficient doses of GK radiosurgery.

## Data Availability

All the data from in this investigation can be disclosed after the request from the editor-in-chief, and the datasets used and/or analyzed during the current study available from the corresponding author on reasonable request.

## Abbreviations

GK: Gamma knife
MR imaging: Magnetic resonance imaging
CT: Computed tomography
